# Knowledge-based repositioning of the anti-HCV direct antiviral agent Sofosbuvir as SARS-CoV-2 treatment

**DOI:** 10.1186/s13027-020-00302-x

**Published:** 2020-05-12

**Authors:** Luigi Buonaguro, Franco M. Buonaguro

**Affiliations:** 1 Laboratory of Innovative Immunological Models, Istituto Nazionale per lo Studio e la Cura dei Tumori, “Fondazione Pascale” – IRCCS, Via Mariano Semmola, 1, 80131 Naples, Italy; 2 Laboratory of Molecular Biology and Viral Oncology, Istituto Nazionale per lo Studio e la Cura dei Tumori, “Fondazione Pascale” – IRCCS, Via Mariano Semmola 52, 80131 Naples, Italy

## Abstract

The new human coronavirus named SARS-CoV-2 is a positive-sense RNA virus for which no specific drugs are currently available. A knowledge-based analysis strongly suggests a possible repositioning of the anti-HCV direct antiviral agent (DAA) Sofosbuvir as treatment for SARS-CoV-2. Indeed, the RNA-dependent RNA-polymerases (RdRp) of the two viruses show high sequence and structural homology, supporting the likelihood of binding the Sofosbuvir molecule with similar efficiency. Such a repositioning would allow the containment of the SARS-CoV-2 pandemic and limit the progression of disease to potentially deadly COVID19.

A new human coronavirus named SARS-CoV-2 was identified in several cases of acute respiratory syndrome in Wuhan, China in December 2019 [1, 2]. The transmission pathways of the new coronavirus include direct transmission (coughing, sneezing and inhalation transmission of droplets) and transmission by contact with mucosa [3]. The viral load of SARS-CoV-2 in saliva can exceed 1 × 10^8^ viral copies per milliliter [4] both in symptomatic and asymptomatic positive subjects [5].

Consequently, it is necessary to reduce or block viral replication to avoid the progression of the disease towards the full-blown and potentially lethal form (COVID19), but also to reduce the viral titer and viral shedding through saliva, in symptomatic and asymptomatic infected individuals.

Specific drugs for SARS-CoV-2 are obviously not available. Currently, drugs originally developed for HIV (e.g. lopinavir, ritonavir) are under evaluation on the basis of weak evidences from retrospective analyses suggesting clinical benefit in the treatment of the two previous coronavirus epidemics [6]. Similarly, anti-malaria chloroquine or hydroxychloroquine are tested [7]. The inhibitor of Influenza’s polymerase Favipiravir is currently evaluated in a clinical trial in combination with anti-IL-6 receptor Tocilizumab (NCT04310228). Finally, the inhibitor of Ebolavirus’ polymerase Remdesivir is currently evaluated in two major SIMPLE clinical trials (NCT04292899; NCT04257656) [8]. On Apr. 29, 2020 it was announced that results from the trial NCT04292899 showed clinical improvement for 50 percent of patients in 10 days in the 5-day treatment group and 11 days in the 10-day treatment group (https://www.gilead.com/news-and-press/press-room/press-releases/2020/4/gilead-announces-results-from-phase-3-trial-of-investigational-antiviral-remdesivir-in-patients-with-severe-covid-19). However, a trial conducted in China showed Remdesivir did not improve patients’ condition nor reduced the positivity to virus. Moreover, the drug showed also significant side effects (https://www.ft.com/content/0a4872d1-4cac-4040-846f-ce32daa09d99).

In the search of the potential best candidate drugs to be repositioned, structural analyses comparing target molecules in the different pathogens should be applied in order to guide a knowledge-based decision process [9].

In the specific case of SARS-CoV-2, and in general in the case of RNA viruses, the most specific target is represented by the RNA-dependent RNA-polymerase (RdRp) which is specific to each RNA virus, regardless the polarity of the viral RNA genome [10, 11]. Nevertheless, significant differences are identified between RdRp from positive-sense and negative-sense RNA viruses [12]. The latter observation strongly suggests that repositioning of antiviral drugs should take into consideration the molecular basis of the genomic viral RNA.

SARS-CoV-2 is a positive-sense RNA virus. The only positive-sense RNA virus, for which a very effective drug targeting specifically the RdRp is available and approved world-wide for clinical use, is hepatitis C virus (HCV). In the specific, Sofosbuvir (Sovaldi®; Epclusa® by Gilead) is a direct antiviral agent (DAA) that inhibits the hepatitis C NS5B RdRp protein [13]. Interestingly, it has been already shown to be effective in vitro and in humans for other two different positive-sense RNA viruses, namely Yellow Fever and Hepatitis A virus [14, 15].

The alignment of RdRp sequences from HCV and the three epidemic/pandemic coronaviruses, confirms the high homology and conservation in several residues along the sequence and in particular in the Motif B and C. On the contrary, such homology is almost lost when RdRp sequences from the three epidemic/pandemic coronaviruses are aligned with those from negative-sense RNA viruses, namely Ebola, Influenza, Rabies and Vesicular Stomatitis viruses [16].

The structure modeling shows that RdRp of positive-sense (HCV and SARS-CoV-2) and negative-sense (i.e. Influenza) RNA viruses are significantly different, but they all show the formation of the Motif C β-strand-loop-β-strand structure. However, only the alignment of RdRp structures from the two positive-sense RNA viruses shows a superimposition of the two Motifs C [16].

All these sequence and structural modelling evidences strongly support the concept that the SARS-CoV-2 RdRp is much more similar to the one from HCV than the one from negative-sense Influenza and Ebola RNA viruses. Therefore, repositioning of Sofosbuvir (Sovaldi®; Epclusa® by Gilead), the inhibitor of the HCV NS5B RdRp protein, as antiviral in the treatment of the SARS-CoV-2 infection has an extremely high potentiality of success, as recently postulated by others [17], and is suggested as a potential drug for the treatment of COVID-19 in the very recent EASL-ESCMID position paper [18]. This is further supported by the great diversity between the molecular structure of the Sofosbuvir and the inhibitors of Influenza and Ebola viruses currently evaluated in clinical trials (Fig. 1).

**Fig. 1:**
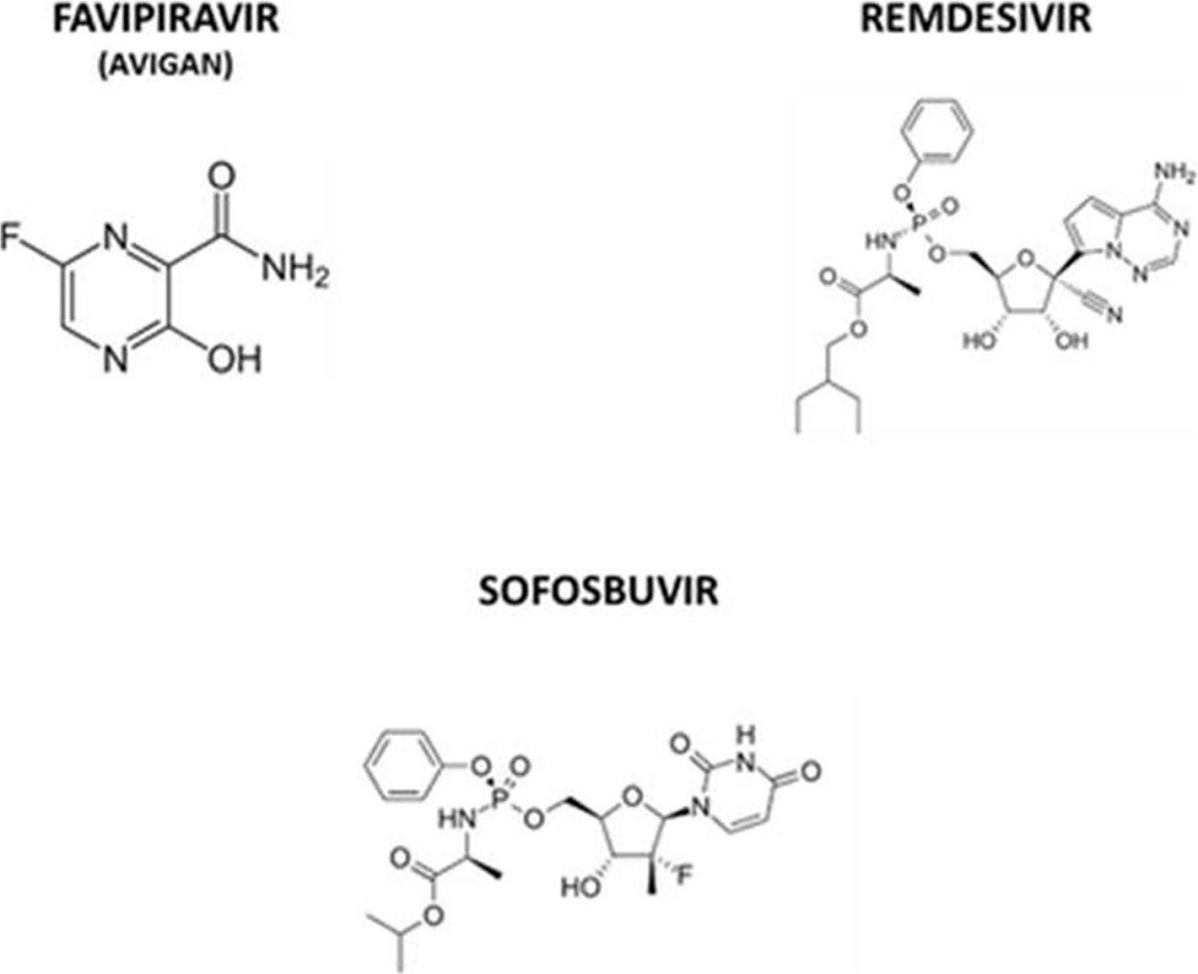
Comparison of RdRp inhibitors of RNA viruses. The structure of the RNA-dependent RNA-polymerase inhibitors in clinical trial, Favipiravir (developed for the influenza virus) and Remdesivir (developed for the Ebola virus) are shown together with Sofosbuvir (developed for the HCV)

Moreover, Sofosbuvir is a prodrug of the protide type and is metabolized to the active antiviral agent GS-461203 (2′-deoxy-2′-α-fluoro-β-C-methyluridine-5′-triphosphate) in the liver. The enzymes involved in such activation are cathepsin A and the histidine triad nucleotide-binding protein 1 (HINT1), and subsequent repeated phosphorylation. All these enzymes have RNA and protein expression in the lung cells comparable to liver cells, ensuring the appropriate activation of the Sofosbuvir to become an effective substrate of the SARS-CoV-2 RdRp in the target cells of the respiratory tract.

In conclusion, the data here reported indicate the Sofosbuvir as the lead compound with the highest potential of antiviral efficacy in SARS-CoV-2 infection. In addition, the long-lasting use in patients for treating and eradicating HCV chronic infection provides also the full knowledge of its safety profile [13]. If the evaluation in humans will hold the premises of the in silico analyses, Sofosbuvir will become an extraordinary therapeutic tool to inhibit the SARS-CoV-2 replication and, ultimately, to make the current pandemic a manageable disease.

## Data Availability

N/A
